# Development of an Artificial Intelligence–Guided Citizen-Centric Predictive Model for the Uptake of Maternal Health Services Among Pregnant Women Living in Urban Slum Settings in India: Protocol for a Cross-sectional Study With a Mixed Methods Design

**DOI:** 10.2196/35452

**Published:** 2023-01-27

**Authors:** Rahul Shrivastava, Manmohan Singhal, Mansi Gupta, Ashish Joshi

**Affiliations:** 1 School of Pharmaceutical and Population Health Informatics Faculty of Pharmacy DIT University Dehradun India; 2 Foundation of Healthcare Technologies Society New Delhi India; 3 School of Public Health University of Memphis Memphis, TN United States

**Keywords:** citizen centric, maternal health, informatics, predictive model, artificial intelligence, development, evaluation, machine learning

## Abstract

**Background:**

Pregnant women are considered a “high-risk” group with limited access to health facilities in urban slums in India. Barriers to using health services appropriately may lead to maternal and child mortality, morbidity, low birth weight, and children with stunted growth. With the increase in the use of artificial intelligence (AI) and machine learning in the health sector, we plan to develop a predictive model that can enable substantial uptake of maternal health services and improvements in adverse pregnancy health care outcomes from early diagnostics to treatment in urban slum settings.

**Objective:**

The objective of our study is to develop and evaluate the AI-guided citizen-centric platform that will support the uptake of maternal health services among pregnant women seeking antenatal care living in urban slum settings.

**Methods:**

We will conduct a cross-sectional study using a mixed methods approach to enroll 225 pregnant women aged 18-44 years, living in the urban slums of Delhi for more than 6 months, seeking antenatal care, and who have smartphones. Quantitative and qualitative data will be collected using an Open Data Kit Android-based tool. Variables gathered will include sociodemographics, clinical history, pregnancy history, dietary history, COVID-19 history, health care facility data, socioeconomic status, and pregnancy outcomes. All data gathered will be aggregated into a common database. We will use AI to predict the early at-risk pregnancy outcomes (in terms of the type of delivery method, term, and related complications) depending on the needs of the beneficiaries translating into effective service-delivery improvements in enhancing the use of maternal health services among pregnant women seeking antenatal care. The proposed research will help policy makers to prioritize resource planning, resource allocation, and the development of programs and policies to enhance maternal health outcomes. The academic research study has received ethical approval from the University Research Ethics Committee of Dehradun Institute of Technology (DIT) University, Dehradun, India.

**Results:**

The study was approved by the University Research Ethics Committee of DIT University, Dehradun, on July 4, 2021. Enrollment of the eligible participants will begin by April 2022 followed by the development of the predictive model by October 2022 till January 2023. The proposed AI-guided citizen-centric tool will be designed, developed, implemented, and evaluated using principles of human-centered design that will help to predict early at-risk pregnancy outcomes.

**Conclusions:**

The proposed internet-enabled AI-guided prediction model will help identify the potential risk associated with pregnancies and enhance the uptake of maternal health services among those seeking antenatal care for safer deliveries. We will explore the scalability of the proposed platform up to different geographic locations for adoption for similar and other health conditions.

**International Registered Report Identifier (IRRID):**

PRR1-10.2196/35452

## Introduction

### Background

The World Health Organization has defined maternal mortality as the annual number of female deaths from any cause related to or aggravated by the pregnancy or its management (excluding accidental or incidental causes) during pregnancy and childbirth or within 42 days of termination of pregnancy, irrespective of the duration and site of the pregnancy, which is estimated to be 295,000 globally each year [1]. Of the total maternal deaths, 94% occur mostly in low-resource settings, which could have been prevented in most cases. In India, there has been a significant decline in maternal deaths in 2015 by 68.7% (n=130 in 2016) against a global maternal mortality rate of 216 (2015) compared with 556 (1990) women dying during childbirth per hundred thousand live births as per the UN Maternal Mortality Estimation Interagency Group report. It has been targeted to reduce below 70 by 2030 under the Sustainable Development Goal [2,3].

There have been various factors contributing to maternal mortality found between the onset of obstetric complications and its outcome. If prompt, adequate treatment is provided, the outcome will usually be satisfactory; therefore, the outcome is most adversely affected by delayed treatment. A review of delayed decisions to seek care and delay in accessing adequate care can provide the actual picture to address the satisfactory utilization of maternal health care services in the context of low- and middle-income countries (LMICs) [4].

There are various national maternal health programs initiated by the Government of India under National Health Mission. Some of them are as follows: (1) Janani Shishu Suraksha Yojana, which is a new approach to health care that emphasizes entitlements and elimination of out-of-pocket expenses for both pregnant women (free delivery including cesarean section in public health facilities) and sick infants [5]; (2) Janani Suraksha Yojna, which provides safe motherhood intervention for reducing maternal and neonatal mortality by promoting institutional deliveries among poor pregnant women by providing conditional cash assistance to below poverty line/schedule caste/schedule tribe women for giving birth in a public health care institution or accredited private hospitals [6]. The Anganwadi center is one of the types of rural childcare service providers in India under the scheme of Integrated Child Development Services, which is one of the flagship programs of the Government of India launched in 1975. It is unique and one of the world’s largest programs for early childhood care and development. With a history of high maternal and infant mortality and morbidity in LMICs like India, the scheme provides preschool nonformal education, on one hand, and breaks the vicious cycle of malnutrition, morbidity, reduced learning capacity, and mortality, on the other, to the beneficiaries including children in the age group of 0-6 years, pregnant women, and lactating mothers [7].

The application of citizen-centric design in this study will primarily keep pregnant women as the beneficiary by understanding user characteristics, needs, and preferences that are critical for developing a human-centered artificial intelligence (AI)–guided predictive model. Analysis of demographic parameters such as gestational age, medical history, immunization, education, and socioeconomic status as well as prior exposure knowledge, practice, and attitude toward the use of antenatal care (ANC) services plays a key role in ensuring optimal use of maternal health services in slums [8].

In urban slums, pregnant women are a “high-risk” group with limited access to health facilities. Barriers to utilization of health services are well documented in a few studies where the urban poor cannot be treated as homogeneous entities because of strong evidence on important sociodemographic variations within the urban poor population with their use of services and the barriers faced in service usage [2,9,10]. On the assessment of social-economic barriers, women reported the poor attitude of health workers. Other barriers reported were men’s stigma-related refusal to accompany their spouses for HIV screening and some bylaws that restrict women who had pregnancy outside marriage to seek an authorization letter from local traditional leaders for ANC services at the health facility [11].

### Rationale of the Study

Most of the urban slums comprise immigrant families bearing their area-specific social customs, rituals, and taboos. Depending on these factors, the slums may differ from one another in their health indicators, which acts as a barrier to timely and appropriate utilization of maternal health care services under the national programs [12,13].

The identified barriers in the utilization of maternal health care services at a local level may differ significantly between slums that need to be recognized, identified, and addressed in the district-level planning for health [14]. Poor families are unable to seek maternal services because of various cultural and demographic factors. The public health care delivery system plays a vital role in the national programs that have been reported to be inefficient; even the minimum facilities are not often made available to the target groups [13,15].

Research shows that AI, automation, and data science can support overburdened health systems and health workers when responsibly deployed. AI-enabled platforms can be used to communicate timely information to the public, support self-care, and communicate initial recommended preventive options and even psychosocial care [16-20]. Hence, we plan to develop an AI-guided citizen-centric predictive model that will help to identify the gaps in using maternal services during ANC and enhance the uptake in utilizing maternal health services during the antenatal period and related pregnancy outcomes.

### Research Hypothesis

The research hypotheses are as follows:

- Is there an association between sociodemographic factors, health behaviors, health care access, clinical factors, and use of maternal health care services such as ANC services?
- Is the design and development of an internet-enabled AI-guided predictive model using a human-centered approach a good fit for enhancing the uptake of maternal health services among pregnant women living in urban slum settings?
- Can the proposed AI-guided predictive model be useful to enhance maternal health services to improve pregnancy-related health outcomes?

### Aims and Objectives

#### Aims

The study has three aims:

1. Identify the factors that affect the uptake of maternal health services such as ANC among pregnant women living in urban slum settings of South Delhi
2. Use community data to design and develop a comprehensive AI-guided citizen-centric tool for improved utilization of maternal health services among pregnant women
3. Evaluate the usefulness and effectiveness of the proposed AI-guided citizen-centric tool to enhance the uptake of maternal health services for ANC services

#### Objective

The objective of our study is to develop and evaluate the AI-guided citizen-centric platform that will support the uptake of maternal health services among pregnant women seeking ANC and living in urban slum settings.

## Methods

### Study Design

A cross-sectional mixed methods study will be conducted to gather both qualitative and quantitative data on an android-based electronic platform. 

### Study Site

The study will be conducted in the urban slums of South Delhi, and participants will be enrolled from the Anganwadi centers. The Anganwadi workers will assist in mobilizing the eligible participants seeking ANC services to participate in the study. 

### Inclusion and Exclusion Criteria

The inclusion criteria are as follows:

- Eligible married pregnant women aged between 18 and 44 years regardless of pregnancy order
- Pregnant women residing in urban slum settings for the last 6 months
- Participants who gave written/e-informed consent
- Participants having a smartphone

Participants who are not willing to participate are not included in the study.

### Study Population

Eligible pregnant women seeking ANC and aged between 18 and 44 years will be enrolled in the study.

### Sample Size

A convenience sampling approach will be employed in selected urban slums across 4 geographic zones of South Delhi where only 10% of the households in each slum site will be selected from each zone. Very few studies reported ANC utilization in urban slums in Delhi. Using a reference of this indicator for urban Delhi considering an expected proportion of 30% of participants completing at least 3 ANC visits, with 95% confidence levels, 6% of absolute precision, and a nonresponse rate of 10%, the estimated sample size of the study will be 225.

### Study Tools

Data collection will be done at the Anganwadi centers using prevalidated instruments adapted from the National Family Household Survey (NFHS). The data will be collected using the electronic data collection tool (Multimedia Appendix 1) focusing on certain predictors that will help in predicting the pregnancy outcomes by measuring the risk associated with the type of delivery method, the term (preterm/full term), and related complications during the delivery. The proposed predictors (Textbox 1) will help in analyzing and comparing the pregnancy outcomes using machine learning from the primary data set.

**Pregnancy predictors to be evaluated in model development: definition.**

**Age**

- Age of the women at the time of data collection during pregnancy

**Duration of pregnancy**

- The current duration of pregnancy from the last menstrual period until the date of data collection

**Education**

- The literacy level of the participant to understand the importance of antenatal care

**Birth order**

- The order a child is born in their family

**Abortion history**

- History of medical termination of pregnancy

**History of diabetes**

- A family history that can make women with a family history of diabetes more at risk for developing the disease

**Hypertension**

- Family history of high blood pressure inducing the pregnancy

**Thyroid**

- Family history of hypo- or hyperthyroidism inducing the pregnancy

**Number of visits**

- Frequency of seeking antenatal care services through Anganwadi centers

**Immunization**

- Immunization against tetanus toxoid during pregnancy

**Supplements**

- Iron folic acid supplements are prescribed during pregnancy

**Ultrasonography**

- Ultrasonography for fetus growth, development, and monitoring of pregnancy

**Related complications**

- Pregnancy-related complications during the prepartum, antepartum, and postpartum period

**COVID-19 history and its related complications**

- History of COVID-19 during pregnancy and its related complications

**Immunization**

- Vaccinations against COVID-19 infection

**Height**

- Height of women during pregnancy at the time of data collection

**Weight**

- Weight of women during pregnancy at the time of data collection about the duration of pregnancy

**Blood pressure**

- Monitoring blood pressure of women during pregnancy

**Pregnancy BMI**

- Body mass divided by the square of the body height

**Knowledge of national maternal and child health programs and benefits**

- Knowledge about various maternal and child health schemes under the Government of India

**Antenatal care accessibility**

- Women seeking antenatal care from health care providers and distance from their residence

### Predictive Model Design

**Figure 1:**
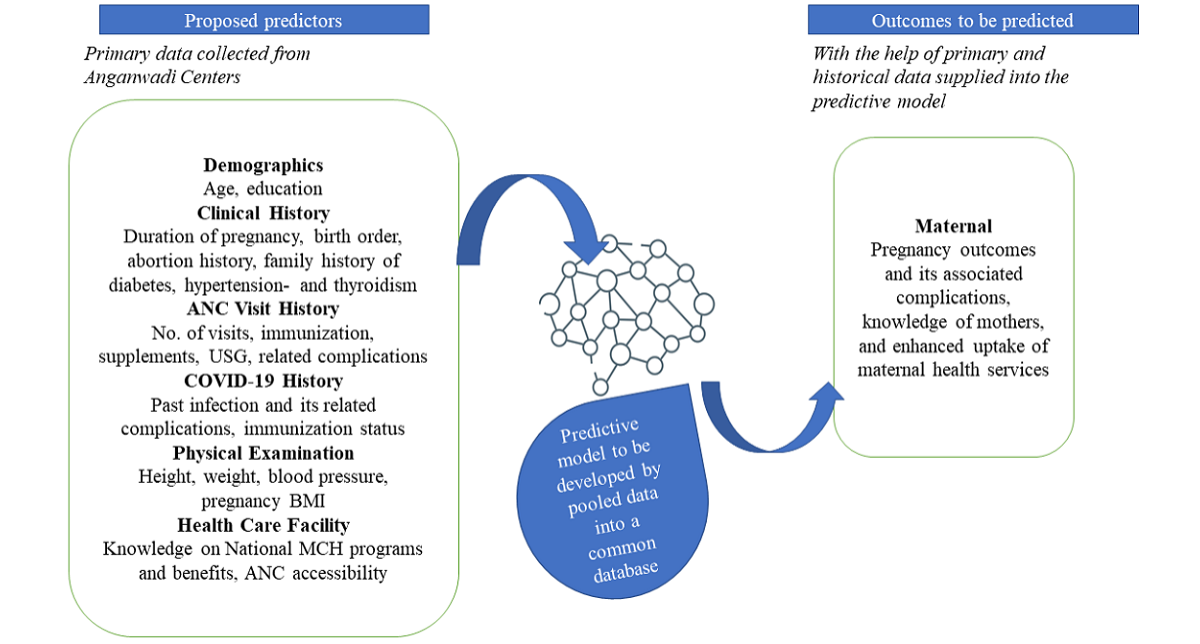
Predictors for comparison in a predictive model of pregnancy outcomes. ANC: antenatal care; MCH: maternal and child health; USG: ultrasonography.

We will develop a machine learning predictive model for early at-risk pregnancy outcomes using citizen-centric design to enhance the uptake of maternal health services among the beneficiaries seeking ANC. This includes some common parameters based on which the model will be developed (Figure 1).

**Figure 2:**
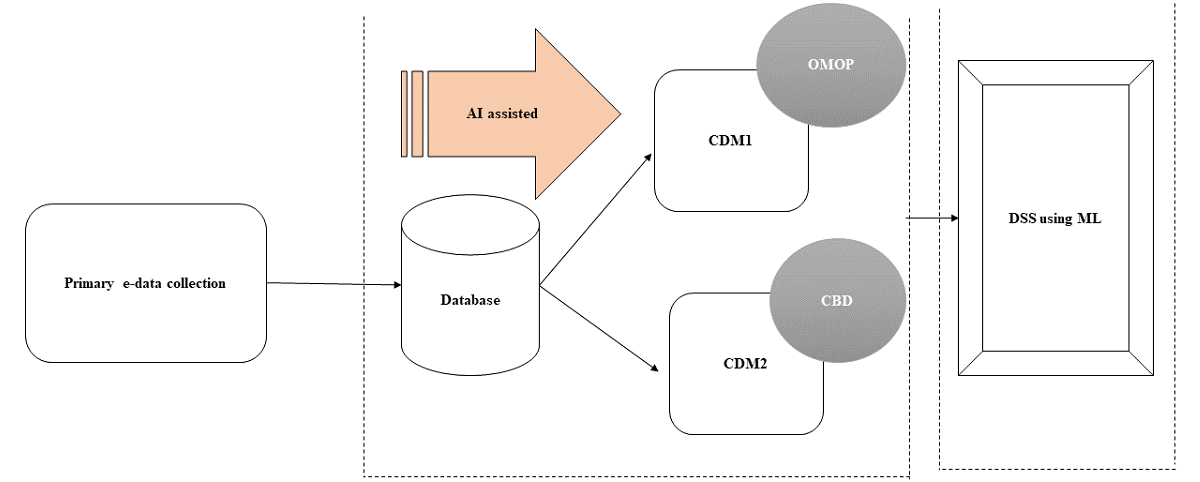
Conceptual framework of data flow to develop the decision support system. AI: artificial intelligence; CBD: community-based demographic model; CDM: common data model; DSS: decision support system; ML: machine learning.

The data pool will be structured following well-established common data models (CDM), which will facilitate harmonizing the data set providing access to relevant data concurrently for ease of consistent and accurate analysis. The CDM, identified at this stage, will include the Observational Medical Outcomes Partnership (OMOP) for clinical data extracted from sources and the customized Community-Based Demographic model for data extracted from communities (Figure 2). The OMOP CDM will allow the organization to standardize medical terms used across the data set for analysis. This platform will focus on algorithm development using evidence-based guidelines [21], existing gaps, and available literature [22,23] to create “IF,” “AND,” and “OR” logics to create a guide for logical thinking and future research, and will help to understand how these data will be processed at the backend, which would help policy makers and other public health researchers in decision-making.

**Figure 3:**
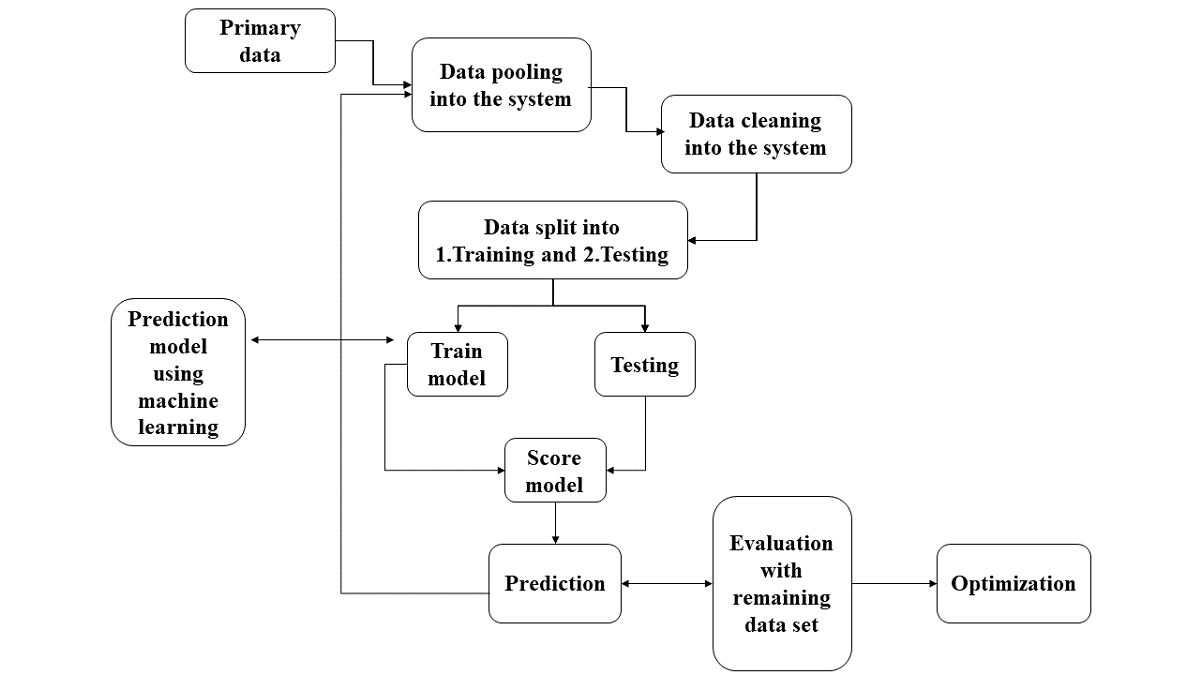
Analytic framework for prediction model of pregnancy outcomes.

The participants will be followed every 3 months from the date of enrollment until their pregnancy outcomes through telephonic calls. The data collected from the first 100 participants will help to develop the AI-guided tool, and the same will be evaluated for its potential competitiveness in the next 125 participants’ data (Figure 3).

### Data Management and Availability

The data will be collected in bilingual languages (English and Hindi) electronically. Source data verification and quality control will be managed by the team of researchers. The anonymized data set for the entire study will be stored in Amazon Web Server (cloud server) and will be used in the model.

### Statistical Analysis

Data will be analyzed using a multivariate regression model that may account for the clustered nature of the pooled data. Uptake in the utilization of maternal health services especially during the antenatal period and pregnancy outcomes (dependent variables: number of ANC visits, tetanus toxoid injections, iron folic acid tablets) will be tested against all independent variables: maternal morbidity, pregnancy outcome, complications of previous pregnancy, literacy, and availability of health care facility using the SPSS statistical tool. Predictive analysis will be compared for accurate future outcomes through the supplied data sets using machine learning to further optimize the model.

### Expected Outcome Measures

The anticipated outcome from this study will be more focused on the epidemiology of maternal health in terms of a better quality of life through the uptake and proper utilization of maternal health services in terms of ANC service utilization efficiently through behavior change communication, advocacy, and health monitoring defining the aspects other than the clinical side in urban slums. Different parameters based on which the efficacy of this proposed platform will be assessed are as follows:

1. Monitoring the trend of the utilization of maternal health services by pregnant women seeking ANC through local service providers (Anganwadi centers) in urban slums
2. Risk identification during the antenatal period
3. Mapping of health care facilities (Anganwadi centers)
4. Evaluating the competencies of the proposed platform in predicting early at-risk pregnancy outcomes.

### Quality Assurance

Three levels of data security will be implemented, namely participant level, data transmission level, and server level, ensuring no harm to study participants (neither physical nor mental) while collecting primary data. For secondary data access from individuals or institutions, prior permissions from respective data owners unless the aforesaid data are in the public domain are obtained (if applicable).

### Ethics Approval

The academic research study has received ethical approval from the University Research Ethics Committee (UREC) of the Dehradun Institute of Technology (DIT) University, Dehradun, India, bearing the approval letter number Protocol DITU/UREC/2021/07/4 dated July 4, 2021. The results will be disseminated at various presentations, seminars, and conferences; as a part of a PhD thesis in Population Health Informatics at DIT University, Dehradun; and in a manuscript that will be submitted to peer-reviewed journals only. The evaluation of the proposed outcome areas will be completed by January 2023.

## Results

The study was approved by the University Research Ethics Committee of DIT University, Dehradun, on July 4, 2021. Enrollment of the eligible participants will begin by April 2022 followed by the development of the predictive model by October 2022 until January 2023. The proposed AI-guided citizen-centric tool will be designed, developed, implemented, and evaluated using principles of human-centered design that will help to predict early at-risk pregnancy outcomes. The results of the study will identify the factors that affect the proper use of maternal health services during the antenatal period. The study results will also intend to make an important contribution by evaluating the usefulness and effectiveness of the proposed predictive model, further evaluating the model for accuracy.

## Discussion

### Principal Findings

In India, slums have been identified in 2613 towns including 20 census towns (19 from the National Capital Territory of Delhi and 1 from Uttar Pradesh) irrespective of their population size in the Census 2011 report, wherein the slum population was enumerated as 22.4% of the total slum population in cities/towns and 17.4% of the total urban population of all the states and union territories [24-27]. The significant community representation from the urban slum population and experience of maintaining population cohorts in the selected study sites are being leveraged to fill knowledge gaps related to the actual burden of maternal morbidity and mortality.

Operational challenges unique to the COVID-19 pandemic, in the form of barriers due to administrative lockdown, restrictions in field mobility in containment zones, and outbreaks at the study site, are anticipated in the study. Hesitance on the part of potential participants toward commonplace data collection at Anganwadi centers and a guarded attitude toward disclosing any health issues due to COVID-19–associated stigma are expected in some communities. We expect that functional linkages with the local health system will be established at each Anganwadi from where the potential participants can be enrolled and benefit from this research study. The study tools adopted from the NFHS will assist in collecting reliable data that are a part of the Indian census enumeration program. The NFHS is a large-scale, multiround survey conducted every 10th year in a representative sample of households throughout India to provide state and national information on fertility, infant and child mortality, the adoption rate of family planning, maternal and child health, reproductive health, nutrition, anemia, and utilization and quality of health and family planning services [28]. Thus, the data collected electronically will thereby help in transforming the raw data into meaningful interpretations and representations for other researchers.

The recruitment sites represent the residents living in the periurban area, generally defined as slum dwellers/*Jhuggi Basti*.

This study will establish the clinical need for a robust prediction model for adverse event–related pregnancy outcomes during the antenatal period to support therapeutic decision-making and continuum of care. Engagement with stakeholders in the model design stage will improve the clinical acceptability of the model and will also support future implementation efforts in the field through the references to a systematic review.

There are a few limitations of this study, such as difficulty in enrollment due to the COVID-19 pandemic, the potential loss to follow-up given the current increase in COVID-19 cases and its complications, participants of the first trimester to be followed until the third trimester down the line to strengthen the system, and results cannot be generalized as the study is limited to one geographic setting with a small sample size.

In this study, no possible risk is anticipated as of now other than the exposure of participants to an infected individual who could be a point of transmission for COVID-19 (considering mega vaccination drives in India) and the emergence of a new variant (Omicron) in the community.

The design of the study will enable us to understand the dynamics of the community, household, and individual due to the inclusion of basic demographic parameters and the socioeconomic status of the family, which will help to analyze the root cause of the underuse of maternal health services and leading complications related to pregnancy outcomes. Subsequent follow-up with the participants will help to evaluate and optimize the model.

### Conclusions

The proposed internet-enabled AI-guided predictive model will help to identify the potential risk associated with pregnancies and enhance the uptake of maternal health services among those seeking ANC for safer deliveries. This study will help to get an insight into various sociodemographic factors other than clinical aspects for underutilization of maternal health services in the community, which will motivate pregnant women to use the government-facilitated national maternal health care program more efficiently, thereby reducing maternal mortality and morbidity during pregnancy. We will explore the scalability of the proposed platform to different geographic locations for use with similar and other health conditions.

## Appendix

Multimedia Appendix 1 Case Record Form.

## Abbreviations

AI: artificial intelligence
ANC: antenatal care
CDM: common data models
DIT: Dehradun Institute of Technology
LMIC: low and middle-income country
NFHS: National Family Household Survey
OMOP: Observational Medical Outcomes Partnership
UREC: University Research Ethics Committee

## Data Availability

For data security, we will implement and maintain appropriate technical and organizational measures following applicable data protection laws to ensure a level of security appropriate to the risks. Information will be collected in an electronic format and will be stored in a secure database. The data will be accessible only to authorized research staff during the study. All the records will be identified with a code and the results/findings may be presented at meetings or in publications; the participant’s identity will not be revealed at any point in time. All possible measures will be taken before the data are made publicly accessible by removing all information that could identify any participant.
