# Supplementary material for: Development of an Artificial Intelligence–Guided Citizen-Centric Predictive Model for the Uptake of Maternal Health Services Among Pregnant Women Living in Urban Slum Settings in India: Protocol for a Cross-sectional Study With a Mixed Methods Design
Source: JMIR Res Protoc. 2023 Jan 27;12:e35452. doi: 10.2196/35452 (PMC9919485; doi:10.2196/35452)
Supplement: Multimedia Appendix 1 [file resprot_v12i1e35452_app1.pdf]

Artificial intelligence guided citizen centric platform to strengthen the uptake of maternal health services amongst the pregnant women living in urban slum settings

Protocol ID	…………………….

Participant ID:	Cluster #	Zone (E/W/N/S)	Slum #	Household #	Serial #

INSTRUCTIONS TO COMPLETE CASE RECORD FORM

1. Read the instructions carefully and fill the form in English only.
2. Complete the source document in black ball point pen.
3. Do not practice the following: - Use of correction fluid
   - Overwriting to make corrections
   - Recording and erasing by pencil
4. Tick (√) to select the response.
5. In case correction is required, strike off the entry using a single horizontal line and re-enter the correct description. Sign and date against the new description. E.g. Date: ~~01 january 2021~~ 01 Jan 2021 (Sign and date)
6. **Participant ID:** Unique identification number allotted to each participant included in the study. This is important and mandate to fill as every record will be linked to this ID.

**Participant ID:**	Cluster #	Zone (E/W/N/S)	Slum #	Household #	Serial #

[***Cluster No.*** *= Assigned no. of cluster for in each zone);* ***Zone*** *= Based on geographical location of urban slums under each cluster (East/West/North/South);* ***Slum No.*** *= Assigned no. urban slum in each cluster;* ***Household No.*** *= Assigned no. of household in each slum;* ***Serial No.*** *= Assigned no. of participant in each household. For Example,* ***Participant ID*** |***01*** |***E*** |***03*** |***05*** |***10*** *(03- Serial no. of the Slum with the name in the list)]*

7. Fill the date in format DD/Mmm/YYYY. e. g. 01/Jan/2021

   If the date is not known, write the month and year. In such cases, fill the date as "Not Known" or NK e.g. nk/Mmm/YYYY. If the month is also not known, fill as nk/nk/YYYY.
8. Fill the time in 24 hours format HH:MM. e. g. 5.10 pm as 17:10
9. If ancillary pages are used, kindly give appropriate reference at relevant visit page.
10. Avoid use of uncommon abbreviations.
11. Answer every question clearly; do not use ditto marks.
12. Do not leave any question unanswered.

केस रिकॉर्ड फॉर्म पूरा करने के निर्देश

1. निर्देशों को ध्यान से पढ़ें और केवल अंग्रेजी में फॉर्म भरें।
2. ब्लैक बॉल पॉइंट पेन में सोर्स डॉक्युमेंट को पूरा करें।
3. निम्नलिखित अभ्यास न करें: - सुधार तरल पदार्थ का उपयोग करें
   - सुधार करने के लिए ओवरराइटिंग
   - रिकॉर्डिंग और पेंसिल से मिटाना
4. प्रतिक्रिया का चयन करने के लिए टिक (√) करें।
5. यदि सुधार की आवश्यकता है, तो एक क्षैतिज रेखा का उपयोग करके प्रविष्टि को काट दें और सही विवरण फिर से दर्ज करें। कृपया नए विवरण के सामने हस्ताक्षर करें और तारीख दें। उदाहरण के लिए तिथि: ~~01 january 2021~~ 01 Jan 2021 (हस्ताक्षर और तिथि)
6. **प्रतिभागी आईडी:** अध्ययन में शामिल प्रत्येक प्रतिभागी को विशिष्ट पहचान संख्या आवंटित की जाएगी। यह महत्वपूर्ण है और भरना अनिवार्य है क्योंकि प्रत्येक रिकॉर्ड इस आईडी से जुड़ा होगा।

**प्रतिभागी आईडी**	Cluster #	Zone (E/W/N/S)	Slum #	Household #	Serial #

*{**क्लस्टर नं.** = नियत सं. प्रत्येक क्षेत्र में क्लस्टर के लिए; **जोन** = प्रत्येक क्लस्टर (पूर्व/पश्चिम/उत्तर/दक्षिण) के तहत शहरी मलिन बस्तियों की भौगोलिक स्थिति पर आधारित; **स्लम नंबर**.= नियत सं. प्रत्येक क्लस्टर में शहरी स्लम; **घरेलू नं.** = नियत सं. प्रत्येक झुग्गी बस्ती में घर का; **क्रमांक** = नियत संख्या प्रत्येक घर में सहभागी की। उदाहरण के लिए, **प्रतिभागी आईडी** / **01** / **E** / **03** / **05** / **10** / (03- **क्रमांक** नं. सूची में नाम के साथ झुग्गी की)]*

7. प्रारूप DD/Mmm/YYYY में तारीख भरें । जैसे 01/Jan/2021
   तारीख का पता नहीं है तो महीने-साल लिख दें। ऐसे मामलों में, तारीख को "ज्ञात नहीं" या एनके जैसे NK/Mmm/YYYY के रूप में भरें। अगर महीने की भी जानकारी नहीं है तो NK/NK/YYYY के रूप में भरें ।
8. 24 घंटे प्रारूप HH:MM में समय भरें. उदाहरण 5.10 बजे के रूप में 17:10 भरें
9. यदि सहायक पृष्ठों का उपयोग किया जाता है, तो कृपया प्रासंगिक यात्रा पृष्ठ पर उचित संदर्भ दें।
10. असामान्य संक्षिप्त रूपों के उपयोग से बचें।
11. प्रत्येक प्रश्न का स्पष्ट उत्तर दें; डिट्टो मार्क्स का प्रयोग न करें
12. किसी भी सवाल को अनुत्तरित न छोड़ें।

Protocol ID	…………………….		Participant ID:	Cluster #	Zone (E/W/N/S)	Slum #	Household #	Serial #

SECTION A – DEMOGRAPHIC DATA/ जनसांख्यिकीय डेटा

Date of screening/ स्क्रीनिंग की तिथि: _ _ / _ _ _ / _ _ _ _ (DD/Mmm/YYYY) **Time/ समय (HH:MM): …… : …….**

Slum Name/ बस्ती का नाम: ………………………………..

Cluster Code/ क्लस्टर कोड: ……….. **Zone/ क्षेत्र: ……………..**

Latitude *(upto 8 decimal)*: **……………………………… Longitude: …………………………..**

**Name of interviewer/ साक्षात्कारकर्ता का नाम**			
**Mode of interview/ साक्षात्कार का तरीका**	**1 = Face to face/ आमने-सामने**<br>**2 = Telephonic/mobile/ टेलीफोनिक/मोबाइल**<br>**3 = Online/ ऑनलाइन**	:	☐

**Respondent's Name/ प्रतिवादी का नाम**		:	
**Respondent Type/ प्रतिवादी प्रकार**	**1 = Self/ स्वयं**<br>**2 = Husband/ पति**<br>**3 = Son/Daughter/ बेटा/बेटी**<br>**4 = Mother/Father/ मां/पिता**<br>**5 = Father/Mother-in-laws/ सास-ससुर**<br>**6 = Brother/Sister/ भाई/बहन**<br>**7 = Grandmother/Father/ दादी/दादा**<br>**8 = Step/Co wife/ सौतेली पत्नी/सह पत्नी**<br>**9 = Other relationship** *(Please specify/* **अन्य संबंध** *(कृपया निर्दिष्ट करें)*<br>**10 = No relation but friends/ servants/ paying guest/ कोई संबंध नहीं बल्कि दोस्त/नौकर/पेइंग गेस्ट**<br>**99 = Don't know/Cannot say/ पता नहीं/नहीं कह सकते**	:	☐<br>………………………………………
**Age** *(in completed years)/* **आयु** *(पूर्ण वर्षों में)*		:	
**Husband/ Father Name/ पति/पिता का नाम**		:	
**Husband/Father Occupation/ पति/पिता का व्यवसाय**		:	
**Participant Occupation/ प्रतिभागी व्यवसाय**		:	

Protocol ID	……………………..		Participant ID:	Cluster #	Zone (E/W/N/S)	Slum #	Household #	Serial #

**Address/ पता**	:	
**Landmark/ सीमा चिन्ह**	:	
**Block/ खंड**	:	
**Slum/ बस्ती**	:	
**Mobile No./ मोबाइल नं.**	:	
**Email Id** *(if available)/* **ईमेल आईडी** *(यदि उपलब्ध हो)*	:	

	**Code/ कोड**		**Response/ प्रतिक्रिया**	**Remarks/ टिप्पणियां**
**Caste/ जाति**	**1 = General/ सामान्य**<br>**2 = Scheduled caste/ अनुसूचित जाति**<br>**3 = Scheduled tribe/ अनुसूचित जनजाति**<br>**4 = Other backward class/ अन्य पिछड़ा वर्ग**<br>**99 = Don't know/ नहीं मालूम**	:	☐	
**Religion/ धर्म**	**1 = Hindu/ हिंदू**<br>**2 = Muslim/ मुसलमान**<br>**3 = Christian/ ईसाई**<br>**4 = Sikh/ सिक्ख**<br>**5 = Buddhist/Neo- Buddhist/ बौद्ध/नव बौद्ध**<br>**6 = Jain/ जैनी**<br>**7 = Jewish/ यहूदी**<br>**8 = Parsi/Zoroastrian/ पारसी**<br>**99 = No religion/ कोई धर्म नहीं**	:	☐	
**Education/ पढ़ाई**	**1 = Profession or Honours/ व्यवसाय या सम्मान**<br>**2 = Graduate/ स्नातक**<br>**3 = Intermediate or diploma/ इंटरमीडिएट या डिप्लोमा**<br>**4 = High School Certificate/ हाई स्कूल प्रमाण पत्र**<br>**5 = Middle School Certificate/ मिडिल स्कूल प्रमाण पत्र**<br>**6 = Primary School Certificate/ प्राथमिक विद्यालय प्रमाण पत्र**<br>**7 = Illiterate/ अशिक्षित**	:	☐	

**Residency Status/ रेजीडेंसी स्थिति**	1 = **Permanent resident** *(Staying in area for more than last 6 months)*<br>**Temporary resident/ अस्थायी निवासी**<br>2 = **Inmigrated for work/employment/ काम/रोजगार के लिए प्रवासित**<br>3 = **Inmigrated for education/ शिक्षा के लिए विस्थापित**<br>4 = **Inmigrated for due to Marriage/ विवाह के कारण प्रवासित**<br>5 = **Inmigrated for Employment/ काम/रोजगार के लिए प्रवासित**<br>6 = **Inmigrated for due to change/ shift of house/ घर के परिवर्तन / शिफ्ट के कारण प्रवासित**<br>7 = **Other** *(please specify)*/ **अन्य** (कृपया निर्दिष्ट करें)	:	☐	
To be filled only when any response to above question is 2,3,4,5,6,7/ केवल तभी भरा जाए जब उपरोक्त प्रश्न का कोई उत्तर 2,3,4,5,6,7 हो				
**Where did you come from? /आप कहां सेआए?**	**State/ राज्य**	:		
	**City/ शहर**	:		
**Occupation/ पेशा**	**1 = Working/ सेवा**<br>**2 = Housewife/ गृहस्वामिनी**	:	☐	
**Do you have any children? / क्या आपके कोई बच्चे हैं?**	**1 = Yes/ हाँ**<br>**2 = No/ नहीं**	:	☐	
**If "Yes", how many children? / यदि "हां", कितने बच्चे?**	**1 = One/ एक**<br>**2 = Two/ दो**<br>**3 = Three/ तीन**<br>**4 = >3 / 3 से अधिक**	:	☐	

Protocol ID	…………………….

Participant ID:	Cluster #	Zone (E/W/N/S)	Slum #	Household #	Serial #

SECTION B – PREGNANCY DATA/ गर्भावस्था डेटा

What was the date of your last menstrual period (LMP)? /आपके पिछले मासिक धर्म (एलएमपी) की तारीख क्या थी?	99 = Don't know/ Can't remember/ पता नहीं/याद नहीं	:	___/_____/______
Duration of pregnancy at present *(In Weeks)*/ वर्तमान में गर्भावस्था की अवधि *(सप्ताह में)*		:	…… Weeks/ सप्ताह
Is this pregnancy registered? क्या यह गर्भावस्था पंजीकृत है?	1= Yes/ हाँ<br>2= No/ नहीं	:	☐
If yes, with whom did you registered? यदि हाँ, तो आपने किसके साथ पंजीकरण कराया?	1= ANM/ ए एन एम<br>2= ASHA/ आशा<br>3= AWW/ आंगनवाड़ी कार्यकर्ता<br>4= Other (*please specify*)/ अन्य (कृपया निर्दिष्ट करें)	:	☐
Did you receive Mother and Child Protection card? क्या आपको मदर एंड चाइल्ड प्रोटेक्शन कार्ड मिला है?	1= Yes/ हाँ<br>2= No/ नहीं	:	☐
Birth Order/ जन्म का क्रम		:	☐
Have you ever adopted family planning? / क्या आपने कभी परिवार नियोजन को अपनाया है?	1 = Yes/ हाँ<br>2 = No/ नहीं	:	
If yes how many months and years ago? / अगर हां कितने महीने और साल पहले?		:	….. Years/ साल<br>…… Months/ महीने
Have you ever/since last visit had one or more pregnancies that resulted in abortion? / क्या आपने कभी/पिछली मुलाकात के बाद एक या एक से अधिक गर्भधारण किए हैं जिसके परिणामस्वरूप गर्भपात हुआ है?	1 = Yes/ हाँ<br>2 = No/ नहीं<br>3 = Not sure/ निश्चित नहीं	:	☐
If yes, what was the reason for abortion? यदि हां, तो गर्भपात का कारण क्या था ?	1= Unplanned pregnancy/ अनियोजित गर्भावस्था<br>2= Contraceptive failure/ गर्भनिरोधक विफलता<br>3= Complication(s) in pregnancy/ गर्भावस्था में जटिलताएं<br>4= Health did not permit/ स्वास्थ्य ने अनुमति नहीं दी<br>5= Female foetus/ कन्या भ्रूण<br>6= Male foetus/ नर भ्रूण<br>7= Economic reason/ आर्थिक कारण<br>8= Last child too young/ पिछला बच्चा बहुत छोटा था	:	☐

	**9= Foetus had congenital abnormality/ भ्रूण में जन्मजात असामान्यता थी**<br>**10= Husband/Mother-in-law did not want/ पति/सास नहीं चाहते थे**<br>**11= Others (*please specify*)/ अन्य (कृपया निर्दिष्ट करें)**		
**Have you ever visited to Antenatal Care? / क्या आपने कभी प्रसवपूर्व देखभाल का दौरा किया है?**	**1 = Yes/ हाँ**<br>**2 = No/ नहीं**	:	☐
**If "Yes", where did you receive antenatal care for this pregnancy? / यदि "हाँ", तो आपको इस गर्भावस्था के लिए प्रसव पूर्व देखभाल कहाँ से प्राप्त हुई?**	HEALTH PERSONNEL/ स्वास्थ्य कार्मिक<br>**1 = Doctor/ डॉक्टर**<br>**2 = ANM/Nurse/ Midwife/LHV एएनएम/नर्स/ मिडवाइफ/एलएचवी**<br><br>OTHER HEALTH PERSONNEL/ अन्य स्वास्थ्य कार्मिक<br>**3 = Dai/ Traditional birth attendant दाई/ पारंपरिक जन्म परिचारक**<br>**4 = Community/ Village heath worker समुदाय/ग्राम स्वास्थ्य कार्यकर्ता**<br>**5 = Anganwadi/ ICDS worker आंगनबाडी/ आईसीडीएस कार्यकर्ता**<br>**6 = ASHA/ आशा**<br>**7 = Other (*Please specify*)/ *अन्य (कृपया निर्दिष्ट करें)***	:	☐
**If "YES" how many times?** *(No. of visits)/* **यदि "हां" कितनी बार?** (यात्राओं की संख्या)		:	☐
**If "NO" please specify reason/ यदि "नहीं" कृपया कारण निर्दिष्ट करें**		:	
**Did you ever receive Tetanus Toxoid (TT) injections? / क्या आपको कभी टिटनेस टॉक्सीइड (टीटी) इंजेक्शन मिले?**	**1 = Yes/ हाँ**<br>**2 = No/ नहीं**	:	☐
**If "Yes" how many? (No. of injections)/ अगर "हां" कितने? (इंजेक्शन की संख्या)**		:	☐

Protocol ID	....

Participant ID:	Cluster #	Zone (E/W/N/S)	Slum #	Household #	Serial #

**Did you ever receive Iron Folic Acid tablets/ syrup? / क्या आपको कभी आयरन फोलिक एसिड की गोलियां/ सिरप मिला है?**	**1 = Yes/ हाँ**<br>**2 = No/ नहीं**	:	☐
**If "Yes" how many days did you take the tablet/ syrup? अगर "हां" तो आपने टेबलेट/सिरप कितने दिनों तक लिया?**		:	☐
**Did you/the pregnant woman have/has an ultrasound test done during this pregnancy? क्या आप/गर्भवती महिला ने इस गर्भावस्था के दौरान अल्ट्रासाउंड परीक्षण किया है?**	**1 = Yes/ हाँ**<br>**2 = No/ नहीं**	:	
**Was/is there any complication during antenatal period? / क्या प्रसवपूर्व काल में कोई जटिलता थी?**	**1 = Antepartum haemorrhage/ एंटेपार्टम नकसीर**<br>**2 = Intrauterine death/ अंतर्गर्भाशयी मौत**<br>**3 = Intra uterine growth retardation/ इंट्रा गर्भाशय विकास मंदता**<br>**4 = Oligohydramnios/ ओलिगोहाइड्रमनियोस**<br>**5 = Increase in blood pressure/ रक्तचाप में वृद्धि**<br>**6 = Increase in blood glucose/ रक्त ग्लूकोज में वृद्धि**<br>**7 = Other (please specify)/ अन्य (कृपया निर्दिष्ट)**<br>**8 = No complications/ कोई जटिलता नहीं**	:	☐<br>..............................

**COVID History/ कोविड परीक्षा**			
**Did you ever have COVID infection in the past? क्या आपको पहले कभी COVID संक्रमण हुआ है?**	**1= Yes/ हाँ**<br>**2= No/ नहीं**	:	
**If "Yes", was this during the pregnancy? / यदि "हाँ", क्या यह गर्भावस्था के दौरान था?**	**1= Yes/ हाँ**<br>**2= No/ नहीं**	:	
**If "Yes", any complication(s) related to pregnancy? / यदि "हां", गर्भावस्था से संबंधित कोई जटिलताएं हैं?**	**1= Yes/ हाँ**<br>**2= No/ नहीं**	:	
**Have you taken COVID-19 vaccine? / क्या आपने COVID-19 का टीका लिया है?**	**1= Yes/ हाँ**<br>**2= No/ नहीं**	:	
**If "Yes", no. of vaccine doses/ यदि "हाँ", टीके की खुराक की संख्या**	**1= First/ पहला**<br>**2= Second/ दूसरा**		

General Examination/ सामान्य परीक्षा		
Present Weight *(in Kg)/ वर्तमान वजन (किलो में)*		…………
Present Height *(in cm)/ वर्तमान ऊंचाई (सेमी में)*		…………
Present Blood pressure *(mmHg)/* वर्तमान रक्तचाप	*Systolic*	…………
	Diastolic	…………
Axillary Temperature (*in* ^{0}*F*)/ एक्सिलरी तापमान *(*^{0}*F में)*		…………

Medical History/ मेडिकल हिस्ट्री				
Present illness/ वर्तमान बीमारी				
History of present illness/ वर्तमान बीमारी का इतिहास				
History of/ का इतिहास -	Diabetes Mallitus/ *मधुमेह मैलिटस*	1 = Yes/ हाँ<br>2 = No/ नहीं	:	☐
	Hypothyroidism/ हाइपोथायरायडिज्म	1 = Yes/ हाँ<br>2 = No/ नहीं	:	☐
	Hypertension/ उच्च रक्तचाप	1 = Yes/ हाँ<br>2 = No/ नहीं	:	☐

Dietary History/ आहार इतिहास				
Questions/ सवाल	Code/ कोड	Responses/ जवाब		Remarks/ टिप्पणियां
Type of Diet/ आहार का प्रकार	1 = Vegetarian/ शाकाहारी व्यक्ति<br>2 = Non-vegetarian/ मांसाहारी<br>3 = Mixed diet/ मिश्रित आहार	:		

Protocol ID	…………………….

Participant ID:	Cluster #	Zone (E/W)	Slum #	Household #	Serial #

**Do you consume Alcohol products? / क्या आप अल्कोहल उत्पादों का उपभोग करते हैं?**	1 = Yes/ हाँ<br>2 = **No/ नहीं**	:	☐	
**Do you consume Tobacco products? / क्या आप तंबाकू उत्पादों का सेवन करते हैं?**	1 = Yes/ हाँ<br>2 = **No/ नहीं**	:	☐	
**If "YES" what form of tobacco? / यदि "हां" तंबाकू का किस रूप में?**	1 = **Smoke Form** (Cigarette/Hookah/Shisha/ E-Cigarette/ Bidi)/ **स्मोक फॉर्म** (सिगरेट/ हुक्का/ शीश/ ई-सिगरेट/ बीड़ी)<br>2 = **Smokeless Form**: (Gutkha/Pan Masala/ Zarda/ Khaini/ Pan Masala) / **धुआं रहितरूप:** (गुटखा/ पान मसाला/ ज़ारडीए खैनी/ पान मसाला)	:	☐	

SECTION C – HEALTHCARE FACILITY DATA/ हेल्थकेयर सुविधा डेटा

Questions/ सवाल	Code/ कोड	Responses/ जवाब		Remarks/ टिप्पणियां
**Did you know about National Maternal Healthcare Services? /क्या आप राष्ट्रीय मातृ स्वास्थ्य सेवाओं के बारे में जानते हैं?**	**1 = Yes/ हाँ**<br>**2 = No/ नहीं**	:	☐	
**Knowledge on Maternal Health Services / मातृ स्वास्थ्य सेवाओं पर ज्ञान**	**1 = Poor/ गरीब**<br>**2 = Medium/ मध्यम**<br>**3 = High/ उच्च**	:	☐	
**Any exposure to media information? / मीडिया की जानकारी के लिए कोई एक्सपोजर?**	**1 = Yes/ हाँ**<br>**2 = No/ नहीं**	:	☐	
**Contact with Female Community Health Volunteer (FCHV) / क्या आपने महिला**	**1 = Yes/ हाँ**<br>**2 = No/ नहीं**	:	☐	

सामुदायिक स्वास्थ्य स्वयंसेवक (FCHV) के साथ संपर्क किया				
Do you believe in Traditional Birth Attenders? / क्या आप पारंपरिक जन्म अटेंडेंट्स में विश्वास करते हैं?	1 = Yes/ हाँ<br>2 = No/ नहीं	:	☐	
Is ANC easily accessible? / क्या ANC आसानी से सुलभ है?	1 = Yes/ हाँ<br>2 = No/ नहीं	:	☐	
If "Yes", how far is ANC located from your home location? *(in kms)*/ यदि "हां", कितनी दूर ANC अपने घर के स्थान से स्थित है? *(किमी में)*		:		
Is necessary information provided by ANC? / क्या एएनसी द्वारा आवश्यक जानकारी प्रदान की गई है?	1 = Yes/ हाँ<br>2 = No/ नहीं	:	☐	
If "No", please specify / यदि "नहीं", तो कृपया निर्दिष्ट करें		:		

SECTION D – SOCIOECONOMIC STATUS/ सामाजिक आर्थिक स्थिति
[MODIFIED KUPPUSWAMY SES 2018] (Indian Journal of Research 2018; 7(3):217-18)

1. **Name of the respondent/ प्रतिवादी का नाम: ………………………………………………………..**
   Relationship with the participant/ प्रतिभागी के साथ संबंध: ………………………………………

2. **Name of the respondent (if any)/ प्रतिवादी का नाम (यदि कोई हो): ……………………………………..**
   Relationship with the participant/ प्रतिभागी के साथ संबंध: ……………………………………………

Information about family members/ परिवार के सदस्यों के बारे में जानकारी:

Name of family members/ परिवार के सदस्यों का नाम	Relationship with participant/ प्रतिभागी के साथ संबंध	Age/ उम्र	Sex/ लिंग	Education/ पढ़ाई	Occupation/ पेशा	Average monthly income/ औसत मासिक आय *(INR)*

Protocol ID	....

Participant ID:	Cluster #	Zone (E/W/N/S)	Slum #	Household #	Serial #

					**TOTAL/ कुल (in INR)**	

**Modified Kuppuswamy Scale for Socioeconomic status assessment – 2018**		
**a) Occupation of Head of the Family** *(Please encircle the appropriate score)*		
Code	***Occupation of the head of the household***	***Score***
**1**	Legislator, Senior Official or Manager	10
**2**	Professionals	9
**3**	Technicians and Associate Professionals	8
**4**	Clerks	7
**5**	Service workers and Shop and Market Sales Workers	6
**6**	Skilled Agricultural and Fishery Workers	5
**7**	Craft and Related Trade Workers	4
**8**	Plant and Machine Operators and Assemblers	3
**9**	Elementary Occupation	2
**10**	Unemployed	1
**b) Education of the Head of the Family** *(Please encircle the appropriate score)*		
Code	***Education of the head***	***Score***
**1**	Profession or Honours	7
**2**	Graduate	6
**3**	Intermediate or diploma	5
**4**	High School Certificate	4
**5**	Middle School Certificate	3
**6**	Primary School Certificate	2

**7**	Illiterate	1
**c) Total Monthly Income of the Family (2018 scale)** *(Please encircle the appropriate score)*		
Code	***Monthly family income in INR***	***Score***
**1**	>126,360	12
**2**	63,182 - 126,356	10
**3**	47,266 - 63,178	6
**4**	31,591 - 47,262	4
**5**	18,953 - 31,591	3
**6**	6327 – 18949	2
**7**	≤6323	1

Total Score = a) + b) + c) = ____________________

Kuppuswamy Socioeconomic Status Scale 2018 *(Tick appropriately)*

**Score**	**Socioeconomic Class**	**Code**	**Response**
26-29	Upper	1	☐
16-25	Upper Middle	2	
11-15	Lower Middle	3	
5-10	Upper Lower	4	
< 5	Lower	5	

SECTION E – FOLLOWUP INFORMATION/ अनुवर्ती जानकारी

**Is follow-up required?** If Yes, fill following details *(Yes=1; No=2)*/ **क्या अनुवर्ती कार्रवाई की आवश्यकता है?** यदि हां, तो निम्नलिखित विवरण भरें *(हां = 1; नहीं =2)*		:	☐
**Status of the Call? / कॉल की स्थिति?**	**1. Answered/ उत्तर**<br>**2. Call connected but not answered/ कॉल कनेक्टेड लेकिन रिप्लाई नहीं किया गया**<br>**3. Switched off/ बंद था**<br>**4. Wrong/discontinued/invalid number/ गलत/बंद/अमान्य नंबर**	:	☐
**Were you able to gather information as per protocol about participant?**	**1. Yes/ हाँ**<br>**2. No (reschedule requested)/ नहीं (पुनर्निर्धारित करने का अनुरोध किया)**<br>**3. No (refused)/ नहीं (मनाकर दिया)**	:	☐

(If 2, fill reschedule details)/ **क्या आप प्रतिभागी के बारे में प्रोटोकॉल के अनुसार जानकारी इकट्ठा करने में सक्षम थे?** *(यदि 2, पुनर्निर्धारित विवरण भरें)*			
**If Re-scheduled/ यदि पुन: शेड्यूल किया जाता है**			
**Reschedule date/ पुनर्निर्धारित तिथि *(DD/Mmm/YYYY)***	**:**	……/……./…………	
**Reschedule time/ समय को फिर से शेड्यूल करें *(HH:MM)***	**:**	…… : ……	
**Pregnancy Outcomes/ गर्भावस्था के परिणाम**		1. Still Birth/ मृतजन्म<br>2. Abortion/ गर्भपात<br>a. Spontaneous abortion/ स्वत: प्रवर्तित गर्भपात<br>b. Induced abortion/ प्रेरित गर्भपात<br>3. Live birth/ जीवित जन्म<br>a. Normal (full term)/ सामान्य जन्म<br>b. Pre-mature (Pre-term)/ असामयिक<br>c. Low birth weight/ जन्म के वक़्त, शिशु के वजन मे कमी होना<br>d. Birth with congenital abnormality/ जन्मजात असामान्यता के साथ जन्म	
